# Prevalence of sight-threatening ocular abnormalities identified by retinal examination in neonates: a systematic review and meta-analysis

**DOI:** 10.1186/s12887-026-07084-y

**Published:** 2026-06-02

**Authors:** Rajendra Prasad Anne, Sheila Samanta Mathai, Shailaja S.

**Affiliations:** 1 Dept. of Neonatology, Kasturba Medical College, Manipal Academy of Higher Education (MAHE), Manipal, Karnataka 576104 India; 2 Dept. of Ophthalmology, Kasturba Medical College, Manipal Academy of Higher Education (MAHE), Manipal, Karnataka 576104 India

**Keywords:** Wide field retinal imaging, Sight-threatening abnormalities, Cataract, Glaucoma, Retinoblastoma, Prevalence

## Abstract

**Background:**

Red reflex testing for newborn screening detects only a fifth of disorders requiring intervention. In this systematic review and meta-analysis, we assessed the prevalence of sight-threatening disorders detected by screening newborn babies with retinal examination.

**Methods:**

We searched PubMed, Embase, Web of Science and Scopus databases for studies assessing newborn retinal examination and reporting treatable sight-threatening abnormalities (cataract, glaucoma and retinoblastoma) and treatable potentially sight-threatening abnormalities (retinitis, vitreous hemorrhage, ROP-like retinopathy and familial exudative vitreoretinopathy). We excluded studies on preterm neonates reporting only ROP and on term neonates reporting only retinal hemorrhages. We adhered to MOOSE guidelines and used the JBI tool for quality assessment and R software for analysis (double arcsine transformation model).

**Results:**

Thirteen observational studies (250,513 neonates) published between 2015 and 2024 were included in the meta-analysis. The studies were from China, India, Brazil, Malaysia, Indonesia, Turkey and New Zealand. Eleven studies used wide-field retinal imaging by RetCam and its variants, and one study each used indirect ophthalmoscopy and smartphone-based fundus imaging devices. On quality assessment, five studies were at increased risk of bias due to small sample size and/or not including consecutive births. The prevalence of treatable sight-threatening abnormalities was 6.1 per 10,000 neonates (13 studies, 250,513 neonates, 95% CI: 1, 14.1), and the prevalence of treatable potentially sight-threatening abnormalities was 42.2 per 10,000 neonates (12 studies, 250,167 neonates, 95% CI: 19.8, 71.9). No adverse effects were reported.

**Conclusions:**

Eye screening of neonates with retinal examination can detect several sight-threatening abnormalities amenable to intervention.

**Trial registration:**

PROSPERO database with registration number CRD42023448129.

**Supplementary Information:**

The online version contains supplementary material available at 10.1186/s12887-026-07084-y.

## Background

Newborn screening forms an essential part of comprehensive neonatal care. The introduction of newborn screening enabled us to identify several neonates early in the course of the disease and provide them with therapeutic and/or preventive measures. Initially, the screening was limited to various blood tests (bloodspot) for biochemical, endocrine and hematological conditions [1]. Gradually, hearing screening [2] and screening for congenital heart diseases [3] were introduced. Eye screening, an essential component of newborn screening, is performed by red reflex testing (RRT). In this bedside test, a source of light is shone into the baby’s eyes, and pupil reflections are observed [4]. Red reflex testing is a relatively easy and safe procedure with minimal adverse effects, and it can be performed by a wide range of healthcare personnel, including primary care providers, nurses and midwives.

However, the use of RRT for newborn eye screening is limited by poor sensitivity despite excellent specificity [5]. In a recent diagnostic test accuracy meta-analysis comparing RRT with any ophthalmological examination [5], only 7.5% of all ocular pathologies and 17.5% of those requiring medical or surgical intervention were identified with RRT. Thus, using RRT alone for newborn eye screening may miss a staggering number of ocular pathologies. While some anterior chamber anomalies, such as cataracts, are detected by red reflex testing, several retinal disorders and posterior chamber abnormalities cannot be detected.

Thus, more detailed eye examinations are required to detect all sight-threatening abnormalities. Retinal examination of neonates performed using indirect ophthalmoscopes or a wide field retinal camera can identify posterior chamber abnormalities in addition to anterior chamber abnormalities [6]. Currently, retinal examination is routinely used in premature neonates for timely identification and treatment of retinopathy of prematurity (ROP) [7]. However, it is not standard practice for neonates not at risk for ROP. Recent studies evaluated the role of retinal examination of all newborns (i.e., extended to neonates of all gestational age and birthweight categories) to detect sight-threatening abnormalities [8–12]. These studies found a high prevalence of ocular abnormalities ranging from 4% to 20%. While most abnormalities were retinal hemorrhages of varying severity, certain conditions, such as cataract, glaucoma, retinitis, retinopathies, retinal detachment, vitreous hemorrhage and optic disc disorders, were frequently reported in these studies. Newborns with these abnormalities can benefit from early detection, ophthalmologic and/or systemic evaluation, and early treatment in conditions such as cataracts, glaucoma, ROP-like retinopathy, extensive vitreous hemorrhages and retinitis of infective origin [13].

Despite the benefits of retinal examination, RRT remains the only recommended screening test in newborns worldwide. In a recent systematic review of international guidelines on universal newborn eye screening, most guidelines recommended RRT with an ophthalmoscope and external eye examination in the age group of 0 to 3 months [14]. With this background, we performed a systematic review of observational studies where retinal examination (Intervention) was conducted for universal newborn eye screening (Population) to assess the prevalence of sight-threatening abnormalities requiring medical or surgical intervention (Outcome).

## Methods

This systematic review was conducted following the guidelines published by Munn et al. [15]. The reporting was performed per MOOSE guidelines (Appendix 1) [16]. The protocol was prospectively registered in the PROSPERO database with registration number CRD42023448129 and can be accessed at https://www.crd.york.ac.uk/prospero/#recordDetails.

### Literature search

We searched EMBASE (via Embase.com; from 1980 to August 2025), MEDLINE (via PubMed; from 1966 to August 2025), Web of Science and Scopus databases. The initial search was conducted on 25th October 2024 and was updated on 14th August 2025. The detailed search strategy is shown in Appendix 2. Gray literature was searched through the Google Scholar database.

### Inclusion and exclusion criteria

We included all observational studies in which retinal examination (using wide field retinal imaging or an indirect ophthalmoscope) was performed on neonates to detect sight-threatening abnormalities (confirmed by an ophthalmologist) requiring intervention or evaluation.

We excluded studies predominantly involving preterm neonates and reporting only retinopathy of prematurity. We excluded studies where newborn eye screening was performed by methods other than retinal examination, namely, red reflex examination, refraction testing, optical coherence tomography, etc. We also excluded studies where retinal examination was performed, but sight-threatening outcomes were not reported (e.g., studies reporting only retinal hemorrhage). Conference reviews without formal publication, guidelines, review articles, and letters or correspondences without original data were also excluded.

### Outcomes

The primary outcomes of interest included potentially sight-threatening disorders that require medical or surgical management, such as cataracts, glaucoma, retinoblastoma, vitreous hemorrhage, chorioretinitis, ROP-like retinopathy and familial exudative vitreoretinopathy (FEVR). We also assessed other abnormalities requiring follow-up, such as retinal hemorrhage, retinal exudates, fundus pigmentation abnormalities, morning glory syndrome, and retinal detachment. We subclassified the disorders as treatable sight-threatening abnormalities, i.e., disorders invariably requiring treatment (including cataract, glaucoma and retinoblastoma), and treatable potentially sight-threatening abnormalities, i.e., disorders that are usually self-resolving but may require treatment in some cases (vitreous hemorrhage, retinitis, familial exudative vitreoretinopathy and ROP-like retinopathy). While some of the lesions, such as colobomas of the retina and optic nerve, hypoplasia of the fovea and optic nerve, optic atrophy, retinal dystrophy and morning glory syndrome, can cause vision loss, we did not include them in the meta-analysis because they lack specific treatment. Retinal hemorrhages are amongst the commonest abnormalities noted on neonatal retinal examination, and the presence of foveal involvement (i.e., foveal hemorrhage) can predispose the neonate to amblyopia. As the search was not designed to capture all studies reporting retinal hemorrhages, we included them as secondary outcomes in the meta-analysis.

### Data extraction strategy

Two reviewers (RPA and SSM) independently performed the title and abstract screening and full-text screening. Any disagreements were resolved by mutual discussion or involvement of the third reviewer (SS). The data extraction from the included studies was performed by two reviewers (RPA and SSM) in a blinded manner. Any disagreements were resolved by mutual discussion or involvement of the third reviewer (SS). We extracted the following data: place of the study, setting, study duration, inclusion and exclusion criteria, method of eye examination (retinal imaging technique, personnel, timing, use of mydriatics, speculum, analgesic measures and topical antibiotics), participant characteristics, outcome details, etc. We have not contacted authors for missing data or incomplete information. We considered the dataset to be duplicated when the study duration and place(s) of the study were similar or overlapping. In such cases, we included the study conducted over the most extensive time frame for inclusion in the meta-analysis.

### Quality assessment

We assessed the risk of bias in each study using the Joanna Briggs Institute (JBI) tool for quality assessment of prevalence studies [17]. We assessed the studies in the domains of sample frame, recruitment, sample size adequacy, description of participants, coverage, methods used to identify the outcome, statistical analysis and follow-up rates. We downgraded the quality of a study if the investigators excluded the neonates inappropriately, did not include consecutively born eligible neonates, had an inadequate sample size (< 2,124 based on a prevalence estimate of 0.005 and a precision of 0.003), did not describe the neonatal characteristics in detail, did not use appropriate identification methods (examination of all subjects not performed by sufficiently trained examiners), did not assess important visual outcomes, did not perform appropriate statistical analysis (numerator and denominator were unclear), or had missing data in > 10% of enrolled subjects. We included all eligible studies in the meta-analysis irrespective of quality. The traffic light plot for visualizing the risk of bias was generated using the robvis tool [18].

### Data synthesis and presentation

Meta-analysis of prevalence was performed for each of the sight-threatening visual outcomes, including cataract, glaucoma, retinoblastoma, vitreous hemorrhage, ROP-like retinopathy, and FEVR. We grouped outcomes as “treatable sight-threatening abnormalities” (cataract, glaucoma and retinoblastoma) and “treatable potentially sight-threatening abnormalities” (vitreous hemorrhage, ROP-like retinopathy, FEVR and chorioretinitis) and calculated the pooled prevalence. We used R statistical software (meta package) for analysis [19]. We used a random effects model (*DerSimonian and Laird/DL method*) to account for within-study and between-study variances [20]. As most of the outcomes are relatively uncommon (i.e., < 1 in 100), we used the *Freeman-Tukey double arcsine transformation* model for meta-analysis [21]. We considered the Cochran Q, I^2^ and τ^2^ statistics as quantitative measures of heterogeneity. We measured prediction intervals to assess the variation in effect size across studies [22]. We used forest plots to visually depict heterogeneity by assessing the variability of prevalence estimates and their confidence intervals across the studies [23]. To identify outlying and influential studies, we constructed Baujat plots (X-axis: Cochran Q-test, Y-axis: influence of each study on summary proportion) [24]. When influential outliers (i.e., studies in the upper right quadrant of the Baujat plot) were identified, we further evaluated their impact on the findings of the meta-analysis using leave-one-out estimation [25]. We planned moderator analysis (subgroup analysis) for studies with different sample sizes (low versus high), participant characteristics (inclusion/ exclusion of NICU-admitted neonates) and different sampling techniques used (consecutive versus nonconsecutive) if the number of studies in each subgroup was more than 10.

Publication bias was assessed using funnel plots, Doi plots and their associated Luis Furuya-Kanamori (LFK) indices [26, 27]. An asymmetry in the Doi plot, with either unequal deviation of limbs of the plot from the midpoint or the presence of more studies in one limb compared to the other, is suggestive of possible publication bias. An LFK index greater than 1 quantitatively indicates the presence of publication bias. We used R software to generate these plots.

## Results

**Fig. 1:**
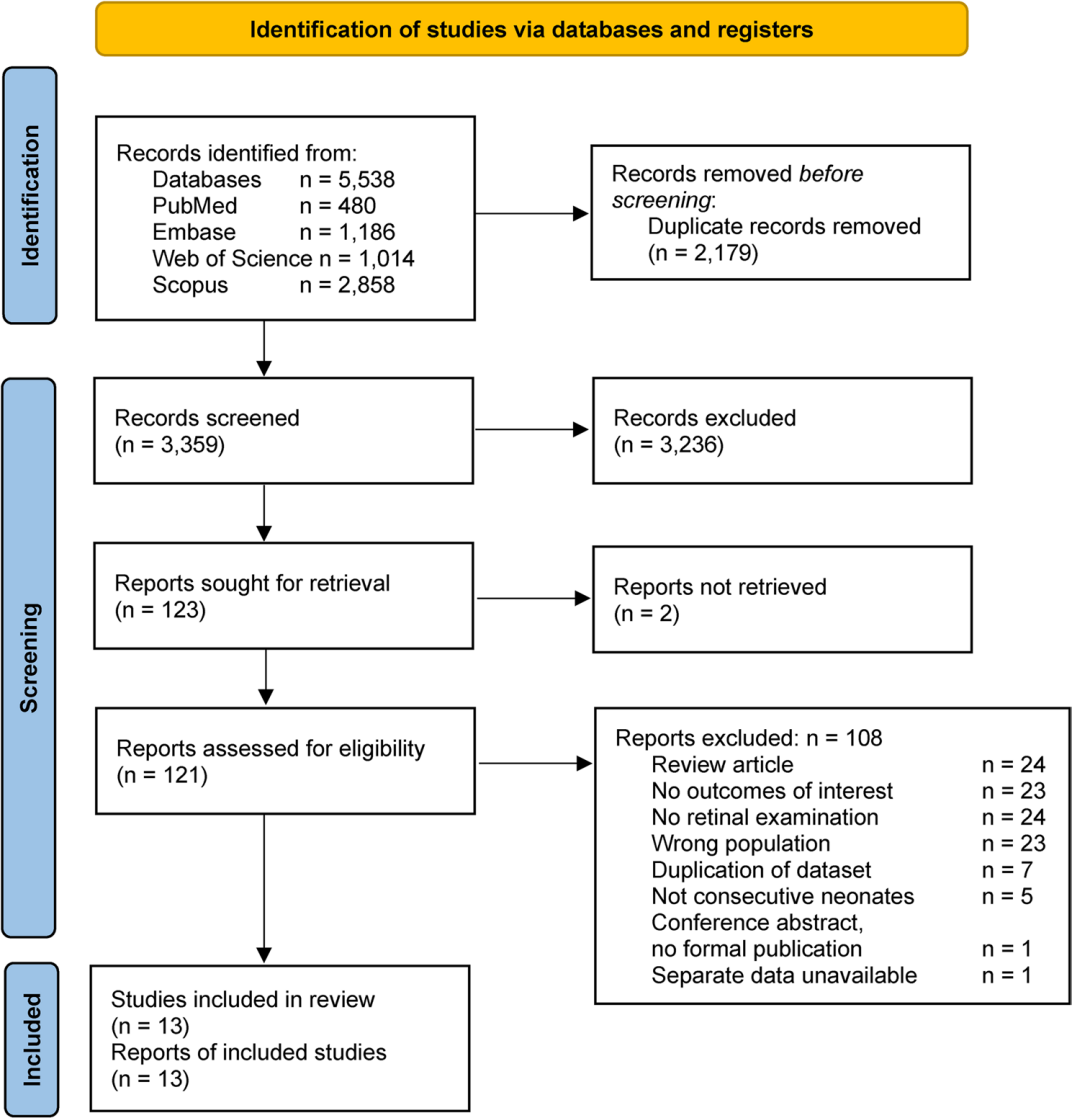
PRISMA flow diagram

We identified 5,538 citations through electronic search and other sources. After removing 2,179 duplicate citations, we evaluated 3,188 studies by reading titles and abstracts. After title and abstract screening, 3,236 studies were excluded because they did not meet the inclusion criteria. Full-text screening was performed for 121 articles (as two could not be retrieved). The details of the 108 excluded studies are provided in the Supplementary Table. Thirteen observational studies were included in the systematic review [8–11, 28–36]. These studies included 250,513 neonates. The PRISMA flow diagram is shown in Fig. 1.

### Study characteristics

The characteristics of the included studies are shown in Table 1. Five studies were from China [9, 28, 30, 33, 35], three from India [8, 11, 34], and one each from Brazil [29], Malaysia [10], New Zealand [31], Indonesia [32] and Turkey [36]. While most studies included stable newborns in maternity wards, two included NICU-admitted babies [28, 35]. In four studies, neonates needing NICU admission were excluded [10, 29, 33, 36]. The inclusion criteria varied from exclusively full-term babies in most studies to babies > 34 weeks/>2000 g [10, 11] to even babies > 30 weeks [31].

**Table 1:** Characteristics of the included studies

Author, year	Country	Duration	Setting	Population	Exclusion criteria
Acar, 2025 [34]	Turkey	October 2021 to October 2023	Ankara Bilkent City Hospital	Full-term newborns with birth weight *≥* 2000 g, post-menstrual age between 36 and 42 weeks and APGAR score *≥* 9	Infants with a history of intensive care unit, infants with anterior segment pathologies
Chen, 2019 [26]	China	January 2016 to May 2018 (29 months)	Daping Hospital, Chongqing	Ward and NICU (Term and preterm)	Poor general condition of baby
da Cunha, 2021 [27]	Brazil	April to June of 2014 (3 months)	Neonates in the Irmandade Santa Casa de Misericordia de Sao Paulo Hospital (ISCMSP) maternity ward.	Infants in the maternity ward	• Admission to intensive care • Additional health conditions which barred their participation in the study • Parent declined participation
Goyal, 2018 [7]	India	March 2014 to Oct 2015 (18 months)	Civil Hospital, Bhubaneshwar	All healthy neonates (within the first 28 days of life) attending the hospital on a fixed day schedule in a week	• Babies older than 4 weeks • Babies too sick to undergo the screening examination (at the discretion of the paediatrician)
Leong, 2021 [9]	Malaysia	January to June 2019 (6 months)	Newborns delivered in the Obstetrics & Gynaecology Labour Room, Hospital Kuala Lumpur (HKL) in Malaysia	Healthy term infants *≤* 28 days, *≥* 2000 g, APGAR *≥* 7. Convenience sampling method used.	Preterm, dysmorphic babies, syndromic babies, congenital diseases, complications intra & postpartum requiring intensive care and monitoring and unstable for an eye examination.
Liu, 2021 [28]	China	January 2017 to July 2020 (43 months)	Ningbo Women’s and Children’s Hospital	Full-term and preterm infants. Data provided separately for term infants	None
Nayak, 2024 [10]	India	June 2016 to April 2018	A government medical college and a private multi-speciality hospital, part of the I-SCREEN project	> 34 weeks and > 2000 g	None
Simkin, 2019 [29]	New Zealand	June 2015 to December 2016 (19 months)	Newborns born in a hospital maternity ward and community delivery centre in the Auckland region	All infants, except those enrolled in ROP screening	Those included in ROP screening (< 1250 or < 30 weeks GA)
Sitorus, 2021 [30]	Indonesia	January 2015 to June 2015 (6 months)	Two hospitals- Cipto Mangunkusumo National Hospital (CM Hospital), the national referral hospital of Indonesia, and Koja Hospital, a district hospital in Jakarta	Healthy, full-term newborn infants aged < 48 h	• The presence of red eyes before the examination • Parents declining to participate in this study
Sun, 2016 [31]	China	September 2014 to March 2015 (7 months)	Jinan Maternity and Child Care Hospital, Jinan	Consecutive newborns born in the hospital	No parental consent, gestational age < 37 weeks, NICU admission, obvious ocular deformity
Tang, 2018 [8]	China	January 2009 to July 2017 (8 years 7 months)	Eight maternal and children’s hospitals (1) Maternal and Children’s Hospital, Liuzhou, Guangxi (2) Maternal and Children’s Hospital, Zhuhai, Guangdong (3) Maternal and Children’s Hospital, Kunming, Yunnan (4) Maternal and Children’s Hospital, Maanshan, Anhui (5) Maternal and Children’s Hospital, Urumqi, Xinjiang (6) Maternal and Children’s Hospital, Huhhot, Inner Mongolia (7) Maternal and Children’s Hospital, Linyi, Shandong and (8) Maternal and Children’s Hospital, Changchun, Jilin	Consecutive newborns born at eight centres across China	None
Vinekar, 2015 [32]	India	September 2012 to March 2013 (7 months)	KC General Hospital, Bengaluru	Neonates born at a public general Hospital and present at the day of screening (thrice weekly)	None
Wang, 2022 [33]	China	December 2018 to December 2021 (48 months)	Newborns admitted to NICU and mother-infant room of Zhongshan People’s Hospital, Zhongshan, Guangdong	Babies in NICU and maternity wards	Not specified

*NICU* Neonatal intensive care unit, *ROP* Retinopathy of prematurity, *GA* Gestational age

The methodological details (choice of mydriatic, topical analgesia, interface, speculum use, eye images obtained, personnel involved and postprocedure antibiotic prophylaxis) are detailed in Table 2. All except three studies [10, 11, 36] used 130-degree wide field retinal imaging (Ret Cam and its variants) for retinal imaging. One study each used a 100-degree retinal camera [10], indirect ophthalmoscope [11] and smartphone-based fundus camera [36]. Mydriatics included various combinations of tropicamide, phenylephrine and cyclopentolate. While ophthalmologists performed eye examinations and retinal image capture in most studies, the images were obtained by optometrists [8], nurse specialists, medical photographers [31], and trained technicians [32, 34] in four studies. In all these studies, the images were verified by an ophthalmologist or a pediatric retina specialist.

**Table 2:** Methodological details

Author, year	Age at exam	Instrument	Mydriasis and analgesia Interface and speculum	Eye images obtained	Personnel	Antibiotics post procedure
Acar, 2025 [34]	First 3 months (85.4% below 1 month)	Smartphone and/or do-it-yourself smartphone-based fundus imaging device with condensing lenses	Phenylephrine 2.5% and tropicamide 0.5% Proparacaine 0.5% Speculum- used	Not specified	Ophthalmologist	Not specified
Chen, 2019 [26]	28–30 days	Retcam III	Tropicamide Proparacaine. Interface and speculum- Not specified	Not specified	Ophthalmologist	Ofloxacin eye ointment
da Cunha, 2021 [27]	Not specified	Not mentioned, 130-degree lens	Tropicamide 0.5% plus phenylephrine hydrochloride 2.5%. Proxymetacaine Sterile paediatric blepharostat, methylcellulose 2% Speculum- not specified	Neonate’s face, anterior segment and red reflex followed by contact photographs of the fundus (optic nerve centred, superior, inferior, nasal, temporal)	One paediatrician- red reflex testing; two ophthalmologists- retinal examination	Not specified
Goyal, 2018 [7]	Not specified	Retcam II, 130-degree lens	Phenylephrine hydrochloride 2.5% plus cyclopentolate 0.5%. Proparacaine Hydrochloride 0.5% Interface- Inert lubricating jelly (K-Y gel) Speculum used	External structures of both eyes, anterior segments of each eye, and fundus imaging. 5 fundus photographs- posterior pole, including disc and fovea, superior retina-optic disc at the inferior pole of the field of view, inferior retina-optic disc at the superior pole of the field of view, temporal-optic disc at the nasal-most part of the field of view and nasal retina- optic disc at the temporal-most part of the field of view. In addition, the superotemporal, inferotemporal, inferonasal, and superonasal quadrant retina were also imaged when required.	The optometrist took images, nurse assisted, and an ophthalmology resident analysed the images; images with positive findings were reevaluated and cross-checked by a pediatric retina specialist	Tobramycin
Leong, 2021 [9]	Within 72 h or at discharge	Phoenix ICON Paediatric Retinal Camera (100 degree view)	Phenylephrine hydrochloride 2.5% plus Tropicamide 1% Tetracaine Hydrochloride 0.5% Interface- Coupling gel Speculum used	Five fundus photographs were taken, and they included the posterior pole (including disc and fovea), superior retina, inferior retina, temporal retina and nasal retina.	Ophthalmology Medical Officer with more than 5 years’ experience	Fusidic acid 1% ointment
Liu, 2021 [28]	Within 30 days	RetCam3 or PanoCam LT	Tropicamide Analgesia was given, but details were not provided Levofloxacin gel Speculum used	Photos were taken of the posterior pole, including the optic nerve and macula. The superior, inferior, nasal, and temporal retinal fields were imaged when possible.	Ophthalmologists	Not specified
Nayak, 2024 [10]	At birth vaccination	Indirect ophthalmoscope	Tropicamide 0.5% plus phenylephrine 2.5%. Proparacaine hydrochloride No interface Speculum used	Not taken	Pediatric Retina Specialist	Not specified
Simkin, 2019 [29]	Not specified	Retcam III or Retcam Shuttle	Tropicamide 1% plus phenylephrine 2.5%. Tetracaine 0.5% Viscotears Liquid Gel (Carbomer 2 mg/g) Speculum used	An anterior image with iris and red reflex; retinal images including the posterior pole with macula and optic nerve, temporal and nasal peripheral retina	Images were captured by a nurse specialist and medical photographer; and remotely reviewed by the specialist paediatric ophthalmologist, using a telemedicine model	Not specified
Sitorus, 2021 [30]	Within 48 h age	Retcam Shuttle	Tropicamide 0.5% plus phenylephrine 2.5%. Analgesia was given, but details were not provided Interface and speculum- not specified	A minimum of five photos were taken for each eye: The posterior pole, superior, inferior, nasal, and temporal retinal fields	Images captured by trained technicians/ 2 general practitioners, assisted by a nurse; Images were analysed by 3 pediatric ophthalmologists	Yes
Sun, 2016 [31]	2–4 days after birth	RetCam III	Tropicamide plus phenylephrine Analgesia was given, but details were not provided Interface not specified Speculum used	Red reflex with an ophthalmoscope, slit-lamp examination, fundus images- peripapillary, temporal, nasal, superior, and inferior fundus	4 pediatric ophthalmologists	Not specified
Tang, 2018 [8]	Within 42 days	Retcam Shuttle	Tropicamide 1% Analgesia not specified Interface and speculum- not specified	The standard external examination, anterior segment examination, RRT and fundus examination; Digital images obtained from the posterior pole, and the superior, nasal and temporal retinal field	Trained ophthalmologists	Not specified
Vinekar, 2015 [32]	Within 72 h	Retcam Shuttle	Cyclopentolate 0.5% plus phenylephrine 2.5% Proparacaine Hydrochloride 0.5% Interface not specified Speculum used	An anterior segment image was obtained without the use of any externally attached lens, and retinal images were obtained using the 130-degree (ROP lens)	Trained technicians obtained images; the Pediatric retina specialist reviewed them within 7 days	Not specified
Wang, 2022 [33]	Not specified	Retcam III	Tropicamide Propemecaine hydrochloride 0.5% Interface not specified Speculum used	Retinal images were collected in order of posterior pole, temporal side, upper side, nasal side and lower side of the fundus optic papilla	Chief Physician of the Ophthalmology department and the senior physician with the title of Attending Physician or higher rank	Tobramycin

*RRT* Red reflex testing, *ROP* Retinopathy of prematurity

**Fig. 2:**
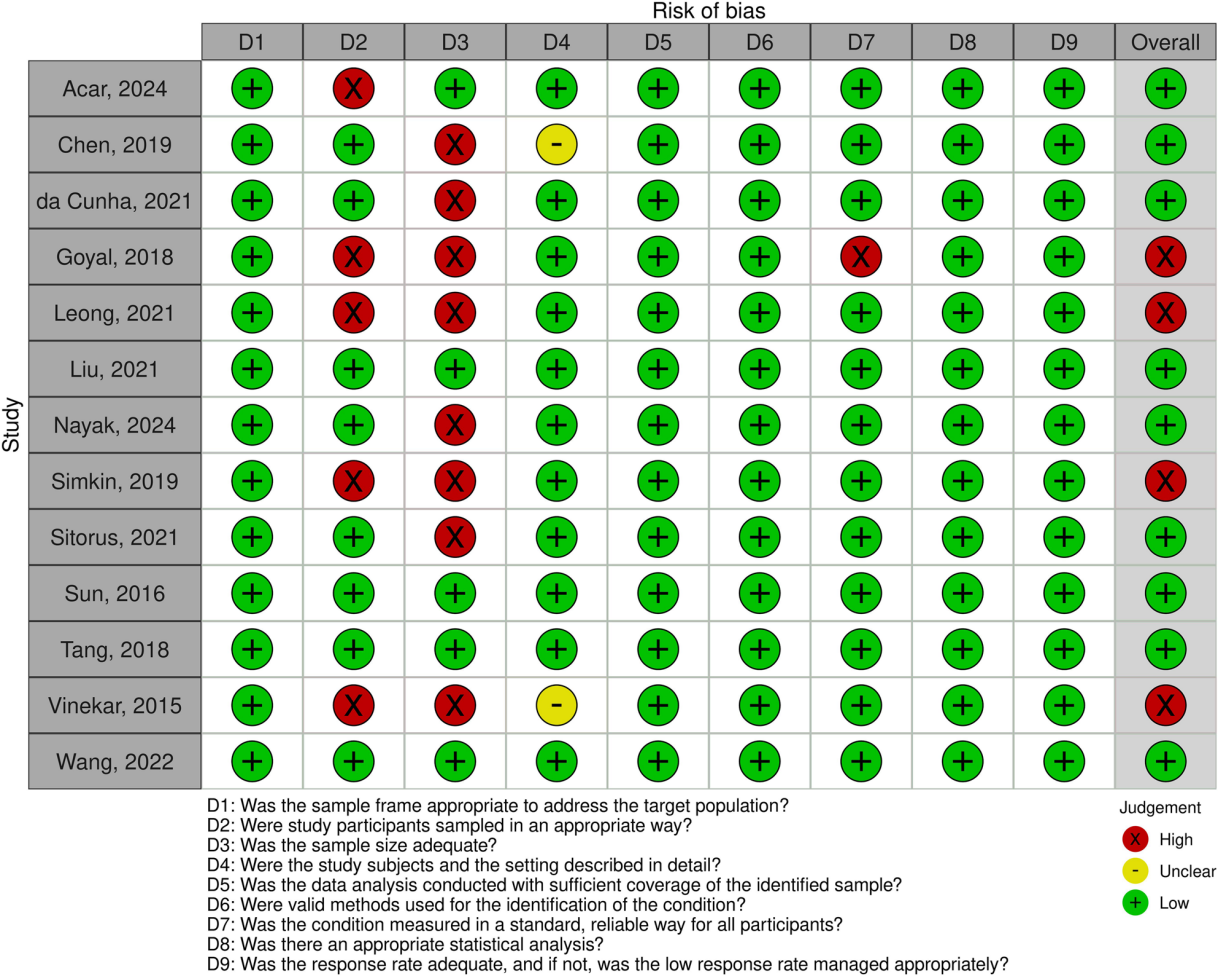
Risk of bias assessment

In the quality assessment, nine studies had a low risk of bias [9, 11, 28–30, 32, 33, 35, 36]. In five studies, the participants were not recruited appropriately (i.e., nonconsecutive samples were included) [8, 10, 31, 34, 36]. Eight studies had inadequate sample sizes [8, 10, 11, 28, 29, 31, 32, 34]. In one study, the assessment of visual conditions was inappropriate, as ophthalmology residents classified the images, and only the abnormal images were viewed by the ophthalmologist [8]. Four studies were rated to be at a high risk of bias because of concerns in two or more domains [8, 10, 31, 34]. The details of the quality assessment are shown in Fig. 2.

### Meta-analysis

**Fig. 3:**
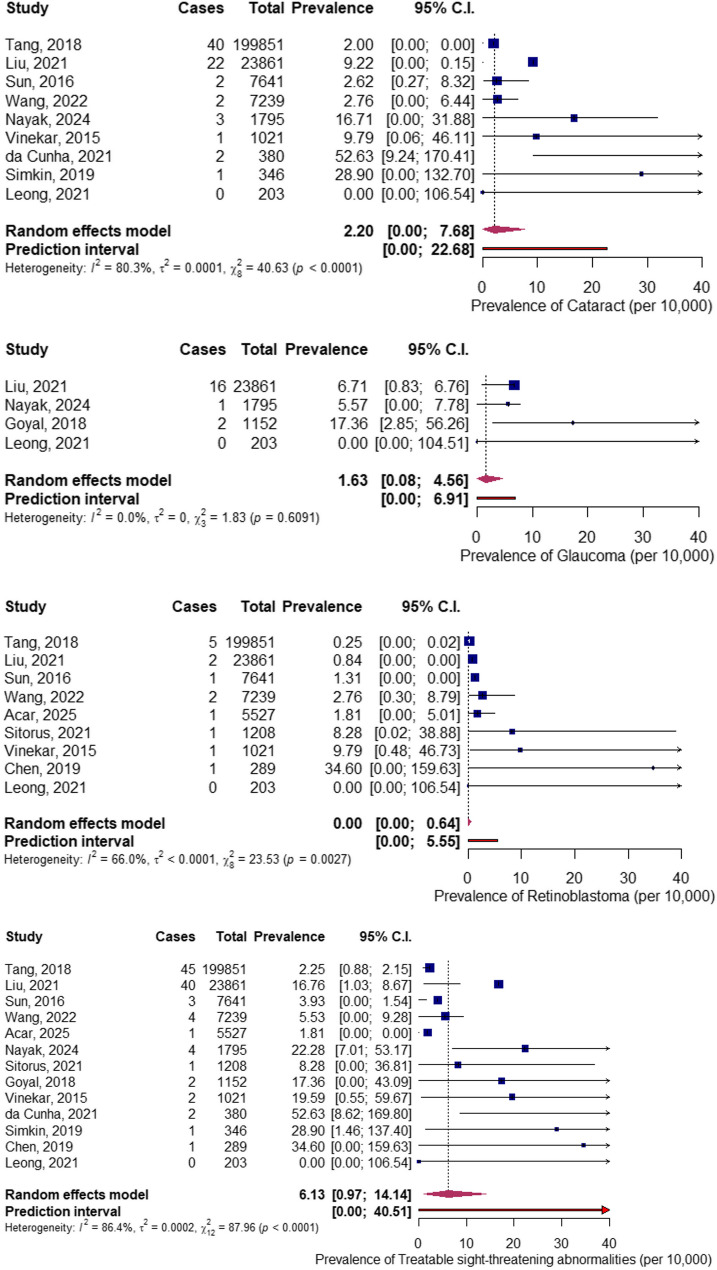
Forest plots showing the prevalence of sight-threatening abnormalities

Among the treatable sight-threatening abnormalities, the prevalence of cataract was 2.2 per 10,000 neonates (9 studies, 242,337 neonates, 95% CI: 0, 7.7), glaucoma was 1.6 per 10,000 neonates (4 studies, 27,011 neonates, 95% CI: 0.1, 4.6), and retinoblastoma was close to 0 per 10,000 neonates (9 studies, 246,840 neonates, 95% CI: 0, 0.6). The cumulative prevalence of treatable sight-threatening abnormalities was 6.1 per 10,000 neonates (13 studies, 250,513 neonates, 95% CI: 1, 14.1), which is approximately 1 in 1,640 neonates. The forest plots are shown in Fig. 3.

**Fig. 4:**
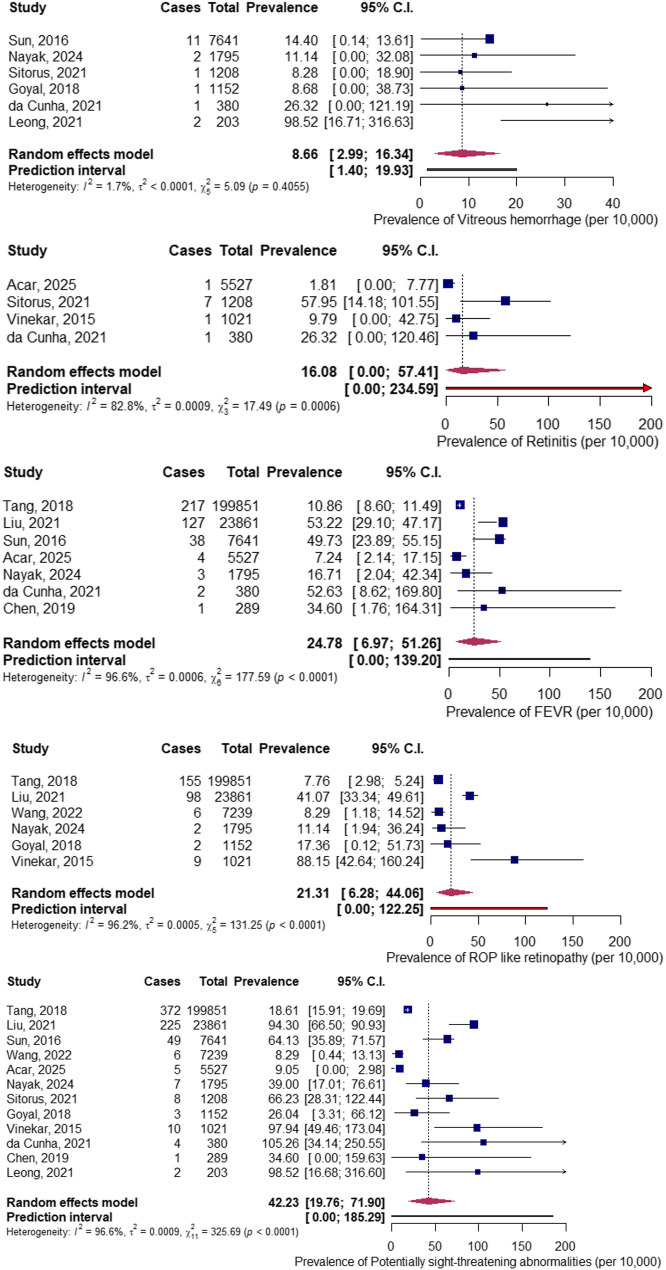
Forest plots showing the prevalence of potentially sight-threatening abnormalities

The prevalence of vitreous hemorrhage was 8.7 per 10,000 neonates (5 studies, 12,379 neonates, 95% CI: 3, 16.3), retinitis was 16.1 per 10,000 neonates (4 studies, 8,136 neonates, 95% CI: 0, 57.4), familial exudative vitreoretinopathy was 24.8 per 10,000 neonates (7 studies, 239,344 neonates, 95% CI: 7, 51.3), and ROP-like retinopathy was 21.3 per 10,000 neonates (6 studies, 234,919 neonates, 95% CI: 19.8, 71.9). The cumulative prevalence of treatable, potentially sight-threatening abnormalities was 42.2 per 10,000 neonates (12 studies, 250,167 neonates, 95% CI: 19.8, 71.9), which is approximately 1 in 237 neonates. The forest plots are shown in Fig. 4.

Two studies provided data on adverse events [28, 33]. None of the neonates had allergic reactions, corneal abrasions, bradycardia or desaturations. The details of other abnormalities detected on screening are shown in Table 3.

**Table 3:** Other abnormalities reported

Author, year	Anterior segment abnormalities
Acar, 2024 [34]	Hypopigmented retinal white lesion- 722, CHRPE- 14, choroidal nevus- 11, idiopathic peripheric retinal scar- 9, choroidal coloboma- 4, retinal coloboma- 4, optic nerve hypoplasia- 2, optic atrophy- 2, retinal calcification- 2, optic nerve pit- 2, MGS- 1, vascular loop on the optic disc- 1, X-linked retinoschisis- 1, thread-shaped white lesion- 1, combined hamartoma of the retina and the RPE- 1, foveal hypoplasia- 1, retinal dystrophy- 1, astrocytic hamartoma 1
Chen, 2019 [26]	MGS- 1, membranous lesion- 1, retinal split- 1
da Cunha, 2021 [27]	Retinal haemorrhages: Sparse- 66 eyes; Dense macular- 38 eyes, dense peripheral- 7 eyes; peri-macular pigmentation- 1, hyphema- 1
Goyal, 2018 [7]	Fundal jaundice- 13, persistent foetal vasculature- 10, foveal hemorrhage- 4, cystic fovea- 3, uveal coloboma- 2 Numbers not provided- sub-conjunctival haemorrhage, vascular dilatation, hypopigmented fundus, immature retina
Leong, 2021 [9]	Subconjunctival haemorrhages- 13, CHRPE– 2, iris naevus- 1
Liu, 2021 [28]	Retinal exudates- 1,590, choroidal coloboma- 42, corneal edema- 24, retinal pigmentation abnormalities- 32, congenital abnormalities of the cornea- 22, persistent foetal vasculature- 11, MGS- 5,
Nayak, 2024 [10]	Zone III avascular retina- 99, Roth spots- 27, zone II avascular retina- 10, endophthalmitis- 4, subconjunctival haemorrhages- 4, iris hypochromia- 1, iris neovascularisation- 1, uveal coloboma- 1, RPE changes at fovea- 1, chorio-retinal atrophic patches- 1, blond fundus- 1, conjunctivitis- 1
Simkin, 2019 [29]	Suspected optic nerve hypoplasia- 2, optic atrophy- 2, choroidal haemangioma- 1, CHRPE- 1
Sitorus, 2021 [30]	Foveal hemorrhage- 4, iris nodule- 1, optic coloboma- 1, persistent pupillary membrane (PPM)- 1
Sun, 2016 [31]	PPM- 217, retinal pigmentation abnormalities- 103, peripheral subretinal exudative change- 51, medullated fibres- 17, abnormal fundus pigmentation- 15, persistent hyaloid artery- 13, vascular abnormalities- 9, hypoplasia or severe cupping- 7, choroidal coloboma- 6, optic atrophy- 2, floaters in aqueous humor- 2, albinotic fundus- 1, persistent foetal vasculature- 1, nebula- 1, MGS- 1, coloboma iridis- 1, nevus of Ota- 1
Tang, 2018 [8]	Abnormal fundus pigmentation- 1,492, retinal exudates- 1,257, abnormal retinal vascularisation- 702, subconjunctival haemorrhage- 256, retinal dysplasia- 193, retinal vascular hypoplasia- 114, PPM- 105, choroidal coloboma- 98, PHPV- 29, other undefined abnormalities- 414
Vinekar, 2015 [32]	Posterior uveitis with linear perivasculitis- 5, retinal dysplasia- 2, peripheral scar- 1, naevus- 1, posterior synechia- 1
Wang, 2022 [33]	Retinal degeneration- 4, corneal leukoplasia- 3, PHPV- 2, PPM- 1, others- 9

*CHRPE* Congenital hypertrophy of retinal pigment epithelium, *MGS* Morning glory syndrome, *PHPV* Persistent hyperplastic primary vitreous, *PPM* Persistent pupillary membranes, *RPE* Retinal pigment epithelium

The details of various conditions requiring treatment, provided by seven studies(8, 9,11 , 30 ,33–35), are shown in Table 4. These disorders included congenital cataracts, congenital glaucoma, retinoblastoma, endophthalmitis, vitreous hemorrhages, cystic fovea, and when progression to neovascularization was noted in conditions such as ROP-like retinopathy, FEVR or zone II avascular retina.

**Table 4:** Treatment and follow-up details:

Author, year	Condition requiring treatment
Goyal, 2018 [7]	• Congenital glaucoma (2 babies): treated appropriately. • Cystic fovea (3 babies): Given topical carbonic anhydrase inhibitors; cysts resolved (by repeat imaging with RetCam) in all within 1–2 months • ROP-like retinopathy (2 babies): evaluated in detail to rule out other ROP mimickers, including FEVR; the ridge regressed with time in one baby, and the other baby was lost to follow-up.
Liu, 2021 [28]	• FEVR complicated with neovascularization (23 babies): retinal laser therapy was performed. • Congenital cataracts, glaucoma, RB, and other diseases that cannot be treated in the index hospital were referred to higher centres.
Nayak, 2024 [10]	• Zone II avascular retina (2 babies): progressed to ROP and required laser. • ROP-like retinopathy (2 babies): required laser for progressive disease (neovascularization) • Endophthalmitis (4 babies): given systemic antibiotics. One infant recovered completely without ocular surgery, while the other three received vitreous biopsy, vitrectomy, lensectomy, and intraocular antibiotics. One eye went into phthisis, while the other three had complete anatomical recovery. The eyes that underwent lensectomy were managed with contact lenses and had intraocular lens implantation 2 years after the primary surgery. • FEVR (3 babies): prophylactic laser therapy • Vitreous haemorrhage (2 babies): early pars plana vitrectomy done to clear the visual axis and prevent amblyopia. • Congenital cataracts (3 babies): Lens aspiration with intraocular lens implantation followed by amblyopia therapy. • Congenital glaucoma (1 baby): Topical dorzolamide for 2 months, followed by trabeculectomy and trabeculotomy for both eyes.
Sun, 2016 [31]	• Congenital cataract (2 babies): no changes at 42 days and three months in 2 cases • FEVR: o 1 baby: received cryotherapy at 5 days age o 3 babies: received anti-VEGF therapy at 4–5 d of age. o Others: followed up • Retinoblastoma (1 baby): transpupillary thermal therapy and chemotherapy at 11 d of age
Tang, 2018 [8]	The babies with abnormal findings (ROP like retinopathy and FEVR) were referred to the ophthalmology department or higher centres for early intervention
Vinekar, 2015 [32]	• Cataract (1 baby): lensectomy was performed • Retinoblastoma (1 baby): treated appropriately • ‘ROP-like ridge’ (9 babies): Over 8 weeks, this resolved in all cases spontaneously • Posterior uveitis (5 babies): evaluated medically • Salt-and-pepper retinopathy (1 baby): evaluated medically • Posterior synechia (1 baby): evaluated medically
Wang, 2022 [33]	• ROP-like retinopathy- o 5 cases: required one-time anti-VEGF injection treatment. o 1 baby: required a two-time anti-VEGF injection (Ranibizumab) • Congenital cataract (2 babies): 1 case was transferred to higher centre; another newborn is being followed up. • Retinoblastoma (2 babies): transferred to higher centres ◊ surgeries were completed.

*ROP* Retinopathy of prematurity, *FEVR* Familial Exudative Vitreoretinopathy, *RH* Retinal haemorrhages, *RB* Retinoblastoma, *VEGF* Vascular endothelial growth factor

The prevalence of retinal hemorrhages was 13 per 100 neonates (12 studies, 250,133 neonates, 95% CI: 8.9, 17) and that of foveal hemorrhages was 34 per 10,000 neonates (2 studies, 2,360 neonates, 95% CI: 10, 57). 

### Heterogeneity assessment

The I^2^ values for glaucoma and vitreous hemorrhage were very low, indicating the absence of heterogeneity for these outcomes. The tau-squared values were close to ‘0’ for all the outcomes, indicating that the true prevalence rates were relatively consistent across the included studies. As the number of studies included was small, we did not consider Cochran’s Q for heterogeneity assessment. The prediction intervals were narrow for the outcomes of cataract, glaucoma, retinoblastoma and vitreous hemorrhages.

### Sensitivity analysis

The Baujat plots (Supplementary Fig. 1) identified outliers for the outcomes of glaucoma, retinoblastoma, treatable sight-threatening abnormalities, retinitis and ROP-like retinopathy. However, in none of the outcomes were the outliers in the upper right quadrant. As these outliers did not significantly contribute to heterogeneity, we did not perform leave-one-out meta-analysis. We did not perform preplanned subgroup analyses as the number of studies in the subgroups based on the covariates (i.e., small versus large sample size, consecutive versus non-consecutive neonates, inclusion/ exclusion of NICU-admitted neonates) was < 10.

### Publication bias

The Doi plots and LFK indices indicated the presence of publication bias for the outcomes studied (Supplementary Fig. 2). In the funnel plots (Supplementary Fig. 3), there was an absence of studies in the lower left quadrant, indicating nonreporting of studies with small sample sizes and/or low prevalence.

## Discussion

In this systematic review and meta-analysis, we assessed the prevalence of sight-threatening abnormalities that can be identified with retinal examination in neonates. We included 13 studies, with 250,513 neonates, conducted in various settings (i.e., low-income, middle-income and high-income countries). We found a significant burden of sight-threatening disorders such as cataract (1 in 4,545 neonates), glaucoma (1 in 6,250 neonates), vitreous hemorrhage (1 in 1,150 neonates), retinitis (1 in 620 neonates), FEVR (1 in 403 neonates), and ROP-like retinopathy (1 in 470 neonates). The cumulative burden of treatable sight-threatening abnormalities was 1 in 1,640 neonates, and treatable potentially sight-threatening abnormalities were 1 in 237 neonates.

The findings of this systematic review and meta-analysis highlight that universal retinal examination of healthy newborns can identify a significant number of abnormalities warranting treatment and/or close monitoring. The burden of treatable diseases identified with retinal examination is comparable to that of disorders such as congenital hypothyroidism (1 in 235 neonates) [37], hearing loss detected by newborn screening (1 in 500 to 1000) [38], and critical congenital heart disease detected by pulse-oximetry screening (1 in 500–800) [39, 40] and much higher than that of disorders such as congenital adrenal hyperplasia (1 in 9,498) [41] and galactosemia (1 in 10,000 to 48,000) [42]. 

When we interpret these findings in light of Wilson and Jungner’s redefined consolidated screening principles [43], universal eye screening of neonates not at risk for ROP fits the criteria for screening. The disease/condition principles (i.e., high incidence, substantial morbidity, well-defined natural history and target population) favor a universal screening program. Similarly, the test principles (accurate, reliable, reproducible, acceptable and interpretable screening test, i.e., retinal examination with recordable photographs, with an agreed management plan for screen-positive neonates. augur well for a screening program. However, the program principles (infrastructure, coordination and integration, acceptability and ethics, benefits and harms, economic evaluation, and quality and performance management) need further evaluation.

Existing WHO guidelines and several other national guidelines recommend universal newborn eye screening by red reflex assessment [14]. In a meta-analysis of five studies, red reflex testing had very poor sensitivity compared to any ophthalmological examination. Only 7.5% of ocular abnormalities and 17.5% of ocular abnormalities requiring medical/surgical intervention were detected [5]. Thus, RRT is not an ideal choice for universal newborn eye screening. Further studies should assess the cost-effectiveness and feasibility of retinal examination of all neonates.

However, implementing retinal examination of all newborns on a large scale can have some challenges. These can be over and above those described for ROP [44–46]. First, there is a deficiency of ophthalmologists trained/interested in newborn screening. While this is a significant challenge, it can be partially overcome by a neonatal-led approach and tele-imaging hub and spoke models tested with ROP screening [47]. In at least four studies, the images were captured by trained technician [32, 34]/ optometrists [8]/ nurse specialist/ medical photographer [31]. Using artificial intelligence models for analyzing retinal images [45] can significantly reduce the burden on ophthalmologists. Second, there may be challenges in covering all newborn babies, especially in low- and middle-income countries with lower institutional births and births in smaller hospitals/clinics with low birth rates. Third, referral pathways will be needed to effectively manage sight-threatening ocular conditions detected by newborn screening.

### Limitations

Although we performed an extensive literature search and adhered to standard guidelines, this systematic review and meta-analysis has a few limitations. Ophthalmologic disorders have not been reported uniformly in the studies. As a result, the estimates of the prevalence of specific conditions may be inaccurate. The quality of studies included in this meta-analysis was limited by a small sample size and the convenience sampling technique in certain studies. Publication bias was noted for most of the outcomes studied. The funnel plots were asymmetrical for most of the outcomes, indicating the possibility of selective reporting of studies with higher prevalence and/or higher sample size. The heterogeneity in the included population (i.e., inclusion of late preterm infants [11] and preterm infants between 32 − 24 weeks [31] in two studies), study setting (inclusion of NICU-admitted babies in two studies [28, 35] and exclusion of such babies [10, 29, 33, 36] in four studies), the timing of the screening test (screening was spanning beyond neonatal age [9, 36] in two studies), the equipment used (indirect ophthalmoscopy [11] and smartphone-based fundus imaging [36] were used in two studies) and the expertise of personnel performing retinal examination could impact the estimates of pooled prevalence. It is worth noting that most of the studies were from developing countries, and thus, the findings of this meta-analysis may not reflect the true global prevalence estimates. Data for various subgroups, such as sick neonates (i.e., those admitted to the NICU), late preterm neonates, low birthweight neonates and small for gestational age neonates, were not available.

## Conclusion

This review indicates that universal eye screening of neonates with retinal examination can detect several sight-threatening abnormalities amenable to intervention. However, the data available are limited, with underrepresentation from several parts of the world. The reporting of the outcomes and the follow-up data were inconsistent. Thus, while retinal examinations have the potential for inclusion in universal newborn screening, there is a need for further studies to assess feasibility and cost-effectiveness before they can be implemented at a larger scale.

## Supplementary Information

Supplementary Material 1.

Supplementary Material 2.

Supplementary Material 3.

Supplementary Material 4.

Supplementary Material 5.

Supplementary Material 6.

Supplementary Material 7.

## Data Availability

All the data used for analysis are provided in tables, figures and supplementary materials.

## Abbreviations

RRT: Red Reflex Testing
PROSPERO: International Prospective Register of Systematic Reviews
MOOSE: Meta-analyses of Observational Studies in Epidemiology
FEVR: Familial exudative vitreoretinopathy
ROP: Retinopathy of prematurity
JBI tool: Joanna Briggs Institute
LFK index: Luis Furuya-Kanamori index
GRADE: Grading of Recommendations Assessment, Development and Evaluation
CI: Confidence interval
NICU: Neonatal Intensive Care Unit
WHO: World Health Organization
