# Supplementary material for: Prevalence of sight-threatening ocular abnormalities identified by retinal examination in neonates: a systematic review and meta-analysis
Source: BMC Pediatr. 2026 Jun 2;26:723. doi: 10.1186/s12887-026-07084-y (PMC13449564; doi:10.1186/s12887-026-07084-y)
Supplement: Supplementary file 1 [file 12887_2026_7084_MOESM1_ESM.docx]

**R meta-analysis results**

**CATARACT**

**pred	ci.lb	ci.ub	pi.lb	pi.ub**

**0.000220 0.000000 0.000768 0.000000 0.001860**

**estimate	ci.lb	ci.ub**

**tau^2	0.0001 0.0001 0.0014**

**tau	0.0104 0.0104 0.0380**

**I^2(%) 80.3095 80.1966 98.1797**

**H^2	5.0786 5.0496 54.9374**

Outliers- None

**GLAUCOMA**

pred	ci.lb	ci.ub	pi.lb	pi.ub

0.000163 0.000008 0.000456 0.000008 0.000456

estimate ci.lb	ci.ub

tau^2	0.0000 0.0000 0.0010

tau	0.0000 0.0000 0.0310

I^2(%)	0.0000 0.0000 88.0709

H^2	1.0000 1.0000 8.3829

**RETINOBLASTOMA**

pred	ci.lb	ci.ub	pi.lb	pi.ub

0.000000 0.000000 0.000064 0.000000 0.000403

estimate ci.lb	ci.ub

tau^2	0.0000 0.0000 0.0010

tau	0.0069 0.0000 0.0318

I^2(%) 65.9967 0.0000 97.6562

H^2	2.9409 1.0000 42.6650

Outliers- Liu and Tang

**SIGHT THREATENING AND TREATABLE CONDITIONS**

pred	ci.lb	ci.ub	pi.lb	pi.ub

0.000613 0.000097 0.001414 0.000000 0.003597

estimate	ci.lb	ci.ub

tau^2	0.0002 0.0001 0.0008

tau	0.0147 0.0084 0.0286

I^2(%) 86.3578 67.3251 96.0142

H^2	7.3302 3.0604 25.0890

Outlier-

**VITREOUS HEMORRHAGE**

pred	ci.lb	ci.ub	pi.lb	pi.ub

0.000861 0.000308 0.001605 0.000305 0.001610

estimate ci.lb	ci.ub

tau^2	0.0000 0.0000 0.0044

tau	0.0005 0.0000 0.0665

I^2(%)	0.1325 0.0000 96.2050

H^2	1.0013 1.0000 26.3505

No outliers identified

**Retinitis**

pred	ci.lb	ci.ub	pi.lb	pi.ub

0.001608 0.000000 0.005741 0.000000 0.012293

estimate	ci.lb	ci.ub

tau^2	0.0009 0.0001 0.0103

tau	0.0299 0.0091 0.1013

I^2(%) 82.8475 30.7694 98.2305

H^2	5.8301 1.4444 56.5122

Acar, 2025 and Sitorus, 2021 are outliers

**FEVR**

pred	ci.lb	ci.ub	pi.lb	pi.ub

0.002478 0.000697 0.005126 0.000000 0.010960

estimate	ci.lb	ci.ub

tau^2	0.0006 0.0001 0.0020

tau	0.0248 0.0094 0.0450

I^2(%) 96.6214 80.2816 98.9485

H^2	29.5981 5.0714 95.1029

No outliers identified

**ROP like retinopathy**

pred	ci.lb	ci.ub	pi.lb	pi.ub

0.002131 0.000628 0.004406 0.000000 0.009111

estimate	ci.lb	ci.ub

tau^2	0.0005 0.0002	0.0040

tau	0.0225 0.0131	0.0630

I^2(%) 96.1904 89.5483 99.4967

H^2	26.2498 9.5678 198.6990

Vinekar, 2015 identified as outlier

After removing outlier:

pred	ci.lb	ci.ub	pi.lb	pi.ub

0.510247 0.506414 0.514079 0.501677 0.518812

estimate	ci.lb	ci.ub

tau^2	0.0002 0.0001	0.0018

tau	0.0156 0.0093	0.0427

I^2(%) 93.6718 83.8806 99.1021

H^2	15.8024 6.2037 111.3701

**POTENTIALLY SIGHT THREATENING Abnormalities**

pred	ci.lb	ci.ub	pi.lb	pi.ub

0.004223 0.001976 0.007190 0.000000 0.016505

estimate	ci.lb	ci.ub

estimate	ci.lb	ci.ub

tau^2	0.0009 0.0003 0.0021

tau	0.0300 0.0167 0.0457

I^2(%) 96.6225 89.8442 98.5203

H^2	29.6079 9.8466 67.5826

No outliers identified
