# Supplementary material for: Prevalence of sight-threatening ocular abnormalities identified by retinal examination in neonates: a systematic review and meta-analysis
Source: BMC Pediatr. 2026 Jun 2;26:723. doi: 10.1186/s12887-026-07084-y (PMC13449564; doi:10.1186/s12887-026-07084-y)
Supplement: Supplementary file 4 [file 12887_2026_7084_MOESM4_ESM.docx]

**Figure 5: Funnel plots**

**Cataract**

**Glaucoma**

**Retinoblastoma**

**Treatable sight threatening abnormalities**

**Vitreous hemorrhage**

**Retinitis**

**Familial exudative vitreoretinopathy**

**ROP like retinopathy**

**Potentially sight threatening abnormalities**
