# Supplementary material for: Prevalence of sight-threatening ocular abnormalities identified by retinal examination in neonates: a systematic review and meta-analysis
Source: BMC Pediatr. 2026 Jun 2;26:723. doi: 10.1186/s12887-026-07084-y (PMC13449564; doi:10.1186/s12887-026-07084-y)
Supplement: Supplementary file 5 [file 12887_2026_7084_MOESM5_ESM.docx]

**Table 2: List of excluded studies**

| **S No** | **Author** | **Year** | **Title** | **Reason for exclusion** |
| --- | --- | --- | --- | --- |
| 1. | Abramov | 1990 | Screening infant vision with paraxial photorefraction | Wrong population |
| 2. | Allingham | 2013 | Racial variation in optic nerve head parameters quantified in healthy newborns by handheld spectral domain optical coherence tomography. | No retinal examination |
| 3. | Amit | 2009 | Vision screening in infants children and youth | Review article |
| 4. | Atkinson | 1983 | THE USE OF ISOTROPIC PHOTOREFRACTION FOR VISION SCREENING IN INFANTS | Review article |
| 5. | Baumann | 1993 | [Prenatal and postnatal determination of gestational age of small newborn infants]. | Review article |
| 6. | Born | 1998 | Visual activation in infants and young children studied by functional magnetic resonance imaging | Wrong population |
| 7. | Cabrera | 2012 | Subfoveal fluid in healthy full-term newborns observed by handheld spectral-domain optical coherence tomography. | No retinal examination |
| 8. | Cabrera | 2013 | Macular findings in healthy full-term hispanic newborns observed by hand-held spectral-domain optical coherence tomography | No retinal examination |
| 9. | Cagini | 2017 | Red reflex examination in neonates: evaluation of 3 years of screening. | No retinal examination |
| 10. | Chee | 2018 | Universal newborn eye screening: An effective strategy to improve ocular health? | Review article |
| 11. | Chen | 2018 | The efficacy and safety of Retcam in detecting neonatal retinal hemorrhages | Duplication of Dataset |
| 12. | Chen | 2018 | The efficacy and safety of Retcam in detecting neonatal retinal hemorrhages | No outcome of interest |
| 13. | Chen | 2011 | Cycloplegic and noncycloplegic refractions of Chinese neonatal infants. | No retinal examination |
| 14. | Cho | 2021 | Birth-related retinal hemorrhages: The Soonchunhyang University Cheonan Hospital universal newborn eye screening (SUCH-NES) study. | No outcome of interest |
| 15. | Cochrane | 2007 | Assessing vision in children | Review article |
| 16. | Costa | 1999 | Early screening of the infants' visual system | No retinal examination |
| 17. | Costenbader | 1968 | Ocular examination before the age of four years. | Wrong population |
| 18. | Critchley | 1968 | Observations on retinal haemorrhages in the newborn | No outcome of interest |
| 19. | de Aguiar | 2011 | Association of the red reflex in newborns with neonatal variables | No retinal examination |
| 20. | de Aguiar | 2007 | [Red reflex: prevention way to blindness in childhood]. | Wrong population |
| 21. | Deng | 2010 | [Refractive error's distribution and clinical characteristics of preterm and full-term children]. | No retinal examination |
| 22. | Duret | 2019 | The infrared reflex: a potential new method for congenital cataract screening. | No retinal examination |
| 23. | Ehlers | 1974 | RETINAL HAEMORRHAGES IN THE NEWBORN- Comparison of delivery by forceps and by vacuum extractor | No outcome of interest |
| 24. | Emerson | 2001 | Incidence and rate of disappearance of retinal hemorrhage in newborns. | No outcome of interest |
| 25. | Eventov-Friedman | 2010 | The red reflex examination in neonates: An efficient tool for early diagnosis of congenital ocular diseases | No retinal examination |
| 26. | Fei | 2021 | Early detection of ocular abnormalities in a Chinese multicentre neonatal eye screening programme-1-year result. | Duplication of Dataset |
| 27. | Feng | 2021 | Comparative study on optic disc features of premature infants and full-term newborns. | No outcome of interest |
| 28. | Fran√ßois | 1982 | Examination of the eye fundus. | Review article |
| 29. | Friedman | 1980 | Ophthalmic screening of 38,000 children, age 1 to 2 1/2 years, in child welfare clinics. | Wrong population |
| 30. | Fung | 2015 | Heidelberg Spectralis Ultra-Widefield Fundus Fluorescein Angiography in Infants | Wrong population |
| 31. | Giles | 1960 | RETINAL HEMORRHAGES IN THE NEWBORN | No outcome of interest |
| 32. | Goyal | 2018 | Outcome of universal newborn eye screening with wide-field digital retinal image acquisition system: a pilot study | Not consecutive |
| 33. | Gursoy | 2018 | The analysis of posterior segment findings in term and premature infants using RetCam images | Not consecutive |
| 34. | Haefeli | 2022 | Portable wide-field digital imaging for screening of neonatal visual impairment causes in Rio de Janeiro, Brazil: A budget impact analysis | No outcome of interest |
| 35. | Hložánek | 2022 | Trends in Neonatal Ophthalmic Screening Methods | Review article |
| 36. | Hodgkinson | 1991 | Characterization of the fundal reflectance of infants | Wrong population |
| 37. | Huang | 2023 | Risk factors of retinal hemorrhage among full-term newborns in Anhui Province of China: a retrospective study | No outcome of interest |
| 38. | Huang | 2023 | Risk factors of retinal hemorrhage among full-term newborns in Anhui Province of China: a retrospective study | No outcome of interest |
| 39. | Hughes | 2006 | Incidence, Distribution, and Duration of Birth-Related Retinal Hemorrhages: A Prospective Study | No outcome of interest |
| 40. | Kandasamy | 2012 | Optic disc measurements in full term infants. | No outcome of interest |
| 41. | Kellner | 2015 | [Inherited retinal or optic nerve disorders ‚Äì five steps to diagnosis]. | Review article |
| 42. | Kharal | 2020 | Flash VEP in clinically stable pre-term and full-term infants | No retinal examination |
| 43. | Khosla | 2005 | Visual screening in newborns, infancy and childhood | Review article |
| 44. | Kogoleva | 2018 | Clinical manifestations and outcomes of retinal hemorrhages in infants: A case series | Wrong population |
| 45. | Laghmari | 2014 | Birth-related retinal hemorrhages in the newborn: Incidence and relationship with maternal, obstetric and neonatal factors. Prospective study of 2,031 cases | No outcome of interest |
| 46. | LaMattina | 2019 | Can the Red Reflex Test Detect Unequal Refractive Error? | Wrong population |
| 47. | Leong | 2021 | Neonatal eye screening for 203 healthy term new-borns using a wide-field digital retinal imaging system | Not consecutive |
| 48. | Li | 2013 | Findings of perinatal ocular examination performed on 3573, healthy full-term newborns. | Duplication of Dataset |
| 49. | Li | 2017 | Full-Term Neonatal Ophthalmic Screening in China: A Review of 4-Year Outcomes | Duplication of Dataset |
| 50. | Li | 2009 | Fundus examination analysis of 313 infants with RetCam II | Wrong population |
| 51. | Li | 2001 | A research of infant refraction in Kunming Municipality | Wrong population |
| 52. | Litmanovitz | 2010 | Red reflex examination in neonates: The need for early screening | Review article |
| 53. | Liu | 2024 | Automated detection of nine infantile fundus diseases and conditions in retinal images using a deep learning system | Duplication of Dataset |
| 54. | Longmuir | 2010 | Nine-year results of a volunteer lay network photo screening program of 147 809 children using a photo screener in Iowa | Wrong population |
| 55. | Ludwig | 2022 | "Higher prevalence of fundus haemorrhages in early-screened (NEST Study) as compared to late-screened (SUNDROP Study) newborn populations" | No outcome of interest |
| 56. | Ma | 2018 | Universal ocular screening of 481 infants using wide-field digital imaging system | Not consecutive |
| 57. | Madan | 2023 | Ophthalmological Surgical Conditions in the Newborn and Neonate | Review article |
| 58. | Magana | 2017 | Use of smartphone fundus camera photographs for screening of retinal pathologies in premature and newborn patients | Conference abstract without formal publication |
| 59. | Mallipatna | 2015 | The use of handheld spectral domain optical coherence tomography in pediatric ophthalmology practice: Our experience of 975 infants and children | Wrong population |
| 60. | Mansoor | 2016 | Eye pathologies in neonates | Review article |
| 61. | Marcou | 2009 | Abnormal ocular findings in the nursery and in the first few weeks of life: a mandatory, yet difficult and neglected screening | Review article |
| 62. | Mohammadzadeh | 2020 | Red reflex examination in neonates for 2 years screening | No retinal examination |
| 63. | Moller | 1980 | Examination of eyes of infants. | Review article |
| 64. | Moreno | 2013 | Choroid Development and Feasibility of Choroidal Imaging in the Preterm and Term Infants Utilizing SD-OCT | No retinal examination |
| 65. | Murooka | 1963 | [DIAGNOSIS OF NEONATAL DISORDERS DUE TO BIRTH]. | No retinal examination |
| 66. | Mussavi | 2014 | The evaluation of red reflex sensitivity and specificity test among neonates in different conditions | No retinal examination |
| 67. | Neme | 1972 | [Effects of assisted labor on the fetal vascular system. I. Examinations of newborn infants by fundoscopy]. | No outcome of interest |
| 68. | Neuweiler | 1967 | Retinah√§morrhagien beim neugeborenen | Review article |
| 69. | Ozkurt | 2018 | Associations between red reflex abnormality, consanguinity and intensive care hospitalization of newborns in Turkey. | No retinal examination |
| 70. | Patel | 2016 | Optic Nerve Head Development in Healthy Infants and Children Using Handheld Spectral-Domain Optical Coherence Tomography | No retinal examination |
| 71. | Patel | 2018 | Ultra-Widefield Imaging for Pediatric Retinal Disease | Review article |
| 72. | Paysse | 2001 | Detection of red reflex asymmetry by pediatric residents using the Br√ºckner reflex versus the MTI photoscreener. | Wrong population |
| 73. | Pu | 2017 | Factors related to retinal haemorrhage in infants born at high risk | No outcome of interest |
| 74. | Remeseiro | 2022 | Case Studies to Demonstrate Real-World Applications in Ophthalmic Image Analysis | Review article |
| 75. | Renz | 1977 | Hyaloid vascular remnants in human neonates | Wrong population |
| 76. | Rodriguez | 1996 | Visual acuity and retinoscopy of preterm and full-term infants during the first year of life | Wrong population |
| 77. | Rothman | 2015 | Assessment of Retinal Nerve Fiber Layer Thickness in Healthy, Full-Term Neonates | No outcome of interest |
| 78. | Sakaria | 2022 | Improving Red Reflex Screening in a Level III NICU Through a Quality Improvement-based Approach | No retinal examination |
| 79. | SanGiovanni | 2002 | Infantile cataract in the collaborative perinatal project: Prevalence and risk factors | Wrong population |
| 80. | Scorolli | 2014 | Prevalence of visual alterations in Italian children using Binocular refractometer and vision analyzer | Wrong population |
| 81. | Scoville | 2022 | Vitreous opacities in infants born full-term and preterm by handheld swept-source optical coherence tomography. | No retinal examination |
| 82. | Semeraro | 2020 | Ocular Refraction at Birth and Its Development During the First Year of Life in a Large Cohort of Babies in a Single Center in Northern Italy | No outcome of interest |
| 83. | Sezen | 1970 | RETINAL HEMORRHAGES IN THE NEWBORN | No outcome of interest |
| 84. | Shafiq | 2015 | Seeing red in young children: The importance of the red reflex | Review article |
| 85. | Sharma | 2020 | MIIRetCam (Make In India Retina Camera) assisted retinal imaging in paediatric patients: Useful, artefacts, learning curve | Review article |
| 86. | Sloot | 2017 | Semistructured Observation of Population-based Eye Screening in The Netherlands | Wrong population |
| 87. | Snir | 2004 | Refraction and keratometry in 40 week old premature (corrected age) and term infants. | No retinal examination |
| 88. | Sut | 2022 | Three-year outcomes of ophthalmological examinations performed at 0–3 months of term/preterm infants | Not consecutive |
| 89. | Szirm√°k | 1971 | [Fundus oculi examinations in premature and mature newborn infants]. | Review article |
| 90. | Tao | 2010 | Study of wide-field digital retinal imaging system on pediatric fundus screening | Wrong population |
| 91. | Tingley | 2007 | Vision screening essentials: Screening today for eye disorders in the pediatric patient | Review article |
| 92. | Tuteja | 2021 | Identification of amblyogenic risk factors with the Br√ºckner reflex test using the low-cost Arclight direct ophthalmoscope | Wrong population |
| 93. | Vehmeijer | 2016 | Retinal Oximetry with Scanning Laser Ophthalmoscope in Infants | No retinal examination |
| 94. | Viquez | 2020 | Sensitivity and specificity of the red reflex in Costa Rican newborns | Review article |
| 95. | Wan | 2015 | Eye disorders in newborn infants (excluding retinopathy of prematurity). | Duplication of Dataset |
| 96. | Wang | 2023 | The ocular anterior segment examination of perinatal newborns by wide-field digital imaging system: a cross-sectional study | Duplication of dataset |
| 97. | Wang | 2018 | Application of a deep convolutional neural network in the diagnosis of neonatal ocular fundus hemorrhage. | No retinal examination |
| 98. | Wasilewski | 2002 | Importance of the ophthalmological evaluation in newborns | Systematic review |
| 99. | Watts | 2013 | Newborn retinal hemorrhages: A systematic review | Separate data for neonates not available |
| 100. | Yan | 2021 | [Analysis of fundus examination results in 8 808 pediatric patients in Northwest China]. | No outcome of interest |
| 101. | Yang | 2022 | Prevalence, Characteristics, and Risk Factors of Retinal Hemorrhage among Full-Term Neonates in Southern China | No outcome of interest |
| 102. | Yanli | 2017 | Risk Factors Affecting the Severity of Full-Term Neonatal Retinal Hemorrhage | No outcome of interest |
| 103. | Yu | 2017 | The evolution of refractive status in Chinese infants during the first year of life and its affected factors | Wrong population |
| 104. | Zhang | 2013 | Clinical application of RetCam II examination in 903 infants | No outcome of interest |
| 105. | Zhao | 2015 | Birth-related retinal hemorrhages in healthy full-term newborns and their relationship to maternal, obstetric, and neonatal risk factors | No retinal examination |
| 106. | Zhi-Gang Xiao | 2013 | Application of RetCam II in the screening  of neonatal fundus disease | Wrong population (OPD based) |
| 107. | Vofo | 2025 | Newborn screening for hearing and sight, Cameroon. | No retinal examination |
| 108. | Bjeloš, M | 2025 | Catching the glow: the power of red reflex screening | Review article |
