# Supplementary material for: Prevalence of sight-threatening ocular abnormalities identified by retinal examination in neonates: a systematic review and meta-analysis
Source: BMC Pediatr. 2026 Jun 2;26:723. doi: 10.1186/s12887-026-07084-y (PMC13449564; doi:10.1186/s12887-026-07084-y)
Supplement: Supplementary file 6 [file 12887_2026_7084_MOESM7_ESM.docx]

**Appendix 2: Search strategy**

| **S No** | **Database** | **Search strategy** |
| --- | --- | --- |
| 1. | **PubMed** | (“Infant, Newborn”[Mesh] OR “Term Birth”[Mesh] OR newborn[tiab] OR neonate [tiab] OR infant [tiab]) AND  (“Neonatal Screening”[Mesh] OR screen*[tiab] OR exam*[tiab] OR test*[tiab] OR assess*[tiab]) AND  (((("Eye"[Mesh]) OR "Retina"[Mesh]) OR "Fundus Oculi"[Mesh]) OR "Optic Nerve"[Mesh] OR ophthal*[tiab] OR ocular[tiab]) AND  (Ophthalmoscopy[Mesh] OR retinoscop*[tiab] OR fundoscop*[tiab] OR “retinal imag*”[tiab]) |
| 2. | **Embase** | ('newborn'/exp OR 'newborn' OR 'infant'/exp OR 'infant') AND ('screening'/exp OR 'screening' OR 'newborn screening'/exp OR 'newborn screening' OR 'examination'/exp OR 'examination' OR 'assessment'/exp OR 'assessment' OR 'testing'/exp OR 'testing') AND ('eye'/exp OR 'eye' OR 'eye fundus'/exp OR 'eye fundus' OR 'retina'/exp OR 'retina' OR 'optic nerve'/exp OR 'optic nerve') AND ('ophthalmoscopy'/exp OR 'ophthalmoscopy' OR 'retinoscopy'/exp OR 'retinoscopy') |
| 3. | **Web of Science** | newborn OR neonate OR infant (Topic) and screen* OR exam* OR test* OR assess* (Topic) and eye OR ophthal* OR ocular OR retina* OR fundus OR ‘optic nerve’ (Topic) and retinoscop* OR ophthalmoscop* OR fundoscop* OR ‘retinal imag*’ (Topic) |
| 4. | **Scopus** | TITLE-ABS-KEY(newborn OR neonate OR full-term OR “full term” OR infant) AND TITLE-ABS-KEY(screen* OR exam* OR test* OR assess*) AND TITLE-ABS-KEY(eye OR ophthal* OR ocular OR retina OR fundus OR “optic nerve”) AND TITLE-ABS-KEY(retinoscop* OR ophthalmoscop* OR fundoscop* OR “retinal imag*”) |
